# Ventral tegmental area disruption in Alzheimer’s disease

**DOI:** 10.18632/aging.101852

**Published:** 2019-03-09

**Authors:** Marco Bozzali, Marcello D’Amelio, Laura Serra

**Affiliations:** 1 Department of Neuroscience, Brighton and Sussex Medical School, University of Sussex, Brighton, East Sussex, United Kingdom; 2 Neuroimaging Laboratory, IRCCS Santa Lucia Foundation, Rome, Italy; 3 Laboratory Molecular Neurosciences, IRCCS Santa Lucia Foundation, Rome, Italy; 4 Unit of Molecular Neurosciences, Department of Medicine, University Campus-Biomedico, Rome, Italy

**Keywords:** VTA, dopamine, connectivity, DMN, fMRI

In the last World Alzheimer Report the global economic impact of Alzheimer’s Disease (AD) and dementias was estimated to be US$818 billion, with 47 million people suffering from AD and representing the 0.5% of the global population [1]. Despite the urgent need for interventions, currently there are no available therapies with the ability to modify the clinical course of AD. No tailored drugs for AD have been approved over the past 15 years, despite massively expensive trials aimed at tackling the disease. An often-cited explanation for the clinical trials’ failure is that they are set too late in the disease course. The cholinergic hypothesis of AD has been extensively investigated and prompted the current use of anticholinesterase inhibitors, whose efficacy is however extremely limited. It has been recently put forward the hypothesis that other neurotransmitter pathways are involved in AD pathophysiology with a special focus on the monoaminergic systems [2–4]. A recent work in a transgenic mice, harbouring human Amyloid Precursor Protein (APP) mutant allele linked to familial AD, demonstrated that degeneration of dopaminergic neurons in the ventral tegmental area (VTA) precedes the deposition of Aβ-plaques in the hippocampus [2]. VTA is a brainstem nucleus containing dopaminergic neurons that project diffusively to the brain cortex by direct and indirect pathways [2]. Interestingly, an association between measures of VTA disruption and memory impairment was previously shown in patients with early AD [3]. The dopaminergic system is known to be implicated in several cognitive and behavioural functions [5] and, importantly, may be modulated by pharmacological interventions [5]. Behavioural symptoms are known to be present since the early clinical stages of AD [6]. They strongly impact on patients’ global disability by causing high distress for the patients themselves and their caregivers. Against this background we recently investigated the potential relationship between disruption of VTA projections and behavioural disorders in patients with AD at different clinical stages [4]. We used functional brain magnetic resonance imaging (MRI) at rest, which has been previously shown to be highly sensitive to AD pathology [7], to selectively investigate the VTA-driven modulation of connectivity in AD brains and its impact on behavioural symptoms. Strikingly, when comparing AD patients to controls, we identified a pattern of disconnection between VTA and the rest of the brain that resembled the so-called default-mode network, with a progressive disruption from early to severe stages of disease. This network, which is known to be selectively targeted by AD pathology, is implicated not only in cognition but also in affective functions, thus accounting for the whole symptomatic spectrum of AD. In our large cohort of patients, the vast majority of them presented with behavioural symptoms regardless of their clinical stage. Among clinical symptoms, the presence and severity of aggression, irritability, and sleep and eating disorders were proven being directly associated to a disruption of VTA connectivity in areas of the brain that are known to be implicated in behaviour. Together with previous findings [2,3], this study further highlights the importance of the dopaminergic system as an early contributor to AD progression with a specific focus on behavioural symptoms. With respect to the neurotransmitters potentially involved in AD pathophysiology, the relationship between cholinergic and dopaminergic dysfunction still needs to be further clarified. It is possible that the involvement of these two pathways is only part of a more complex pathophysiological picture, with individual contributions that may impact differently at different disease stages. This opens novel therapeutic perspectives that need to be explored and tested in clinical trials. Furthermore, this stresses the importance of multidimensional research projects that combine basic/animal models to translational studies in humans.
